# Survey of Barbell Trajectory and Kinematics of the Snatch Lift from the 2015 World and 2017 Pan-American Weightlifting Championships

**DOI:** 10.3390/sports8090118

**Published:** 2020-08-25

**Authors:** Aaron J. Cunanan, W. Guy Hornsby, Mark A. South, Kristina P. Ushakova, Satoshi Mizuguchi, Kimitake Sato, Kyle C. Pierce, Michael H. Stone

**Affiliations:** 1 Center of Excellence for Sport Science and Coach Education, Department of Sport, Exercise, Recreation, and Kinesiology, East Tennessee State University, 1276 Gilbreath Drive, Johnson City, TN 37614, USA; southma@etsu.edu (M.A.S.); ushakovak@etsu.edu (K.P.U.); mizuguchi@etsu.edu (S.M.); jpnsatok@hotmail.com (K.S.); stonem@etsu.edu (M.H.S.); 2 Department of Coaching and Teaching Studies, West Virginia University, 375 Birch Street, Morgantown, WV 26505, USA; william.hornsby@mail.wvu.edu; 3 LSU Shreveport Weightlifting Center for High Performance and Development, Department of Kinesiology and Health Science, Louisiana State University Shreveport; 1 University Place, Shreveport, LA 71115, USA; kyle.pierce@lsus.edu

**Keywords:** biomechanics, technique analysis, Olympic-style lifting

## Abstract

Analysis of elite performances is important to elucidate the characteristics of effective weightlifting technique contributing to the highest level of achievement. The general technique of the weightlifting movements is well established. However, it is also apparent that weightlifting technique can differ based on athlete characteristics. Thus, existing technical models may not accurately reflect current technique of top performers or be applied generically to athletes of different skill, size, sex, or ability. Therefore, the purpose of this descriptive study was to update the scientific knowledge of snatch technique of top international weightlifters. This study used video analysis to determine barbell trajectory and kinematics of 319 successful snatch attempts from two major international competitions. Relative frequencies of barbell trajectory types differed based on competition, sex, category, and ranking. No statistical differences were observed among the top-three performers for either sex for most kinematic variables, and there were no overall discernible patterns of effect size differences for individual or clusters of kinematic variables. The results of this study indicate that weightlifting success can be achieved with a variety of technique profiles.

## 1. Introduction

Attempts completed during major international weightlifting competitions represent maximum or near-maximum performances by the most-skilled performers of the weightlifting movements (the snatch, the clean, and the jerk), and these performances are instructive for the idealization of effective weightlifting technique. The general technique of the weightlifting movements is well established [1,2,3,4,5]. However, it is also apparent that weightlifting technique can differ based on athlete characteristics. For example, differences in the relative frequencies of barbell trajectory types among lifters in A versus B sessions during international competitions have been observed [4,6]. Several authors have also reported differences in lifter and barbell kinematics and kinetics based on skill level [6,7,8]. Other investigations of competitive performances provide evidence that weightlifting technique may also be influenced by other factors such as anthropometry, weight category, and sex [4,6,9,10,11,12]. Such findings may provide some guidance to individualize teaching and coaching to best suit each athlete.

Technique differences may also be suggestive of a need for different training objectives or emphases to address deficiencies that may influence weightlifting success or sport performance. For example, some authors have suggested that the ability to execute a stretch-shortening cycle during the transition phase may be improved by increasing knee flexor concentric strength [6,8] and knee extensor eccentric strength [9,11,13]. Additionally, coaches may employ physical training to increase an athlete’s speed of moving under the barbell after completing the second pull [7].

The best performers may use techniques that are considered suboptimal [9]. However, it is unclear whether top performers with suboptimal technique achieve success because of or despite their technique. Furthermore, the observed technique differences in many of the aforementioned studies are confounded by differences in weightlifting ability (i.e., absolute load lifted), and the technique an individual exhibits is partly dependent on her or his absolute and relative strength. Thus, strength is likely to be a primary determinant of weightlifting success [14].

These technique and strength differences notwithstanding, analyses of performances during major international competitions are infrequent [4,6,9,11,12,15,16,17] or limited to select rankings or weight categories [9,11]. Thus, existing technical models may not accurately reflect current technique of top performers or be generalizable to athletes of different skill, size, sex, or ability. Accurate technical models are important for coaches to devise frameworks for teaching and coaching the weightlifting movements. Technical and biomechanical analyses can also provide rationale for coaches to implement the weightlifting movements in training on the basis of specificity [18,19,20] and transference of training [21,22].

Cross-sectional analyses contribute to the cumulative scientific knowledge on weightlifting technique with the potential to inform coaching practices. Analysis of elite performances is important to elucidate the characteristics of effective weightlifting technique at the highest levels of achievement. Additionally, observed technique differences indicate the need for serial investigations encompassing multiple subgroups of performers. Therefore, the purpose of this descriptive study was to update the scientific knowledge of snatch technique of top international weightlifters by examining barbell trajectory and kinematics of successful snatch attempts by lifters at the 2015 World Weightlifting Championship (WWC) and 2017 Pan-American Weightlifting Championship (PAWC).

## 2. Materials and Methods

### 2.1. Participants

This study was an investigation of the heaviest successful snatch attempt for all athletes who lifted in the A sessions of WWC and PAWC. The heaviest successful snatch attempt by each of the top-three finishers at the time of competition in each women’s and men’s weight category for WWC and PAWC was also identified for separate analyses. Seven women’s and 8 men’s weight categories were contested at WWC, and 8 weight categories each for both women and men were contested at PAWC. Three eligible attempts each from both WWC and PAWC were not recorded due to software/hardware error, which incidentally resulted in the exclusion of the 3rd place finisher in the women’s 75 kg category from WWC and the 3rd place finisher in the women’s 48 kg category from PAWC from the comparative analyses. Seven lifters from WWC and 4 lifters from PAWC did not complete any successful attempts in the snatch and were also excluded from analysis. In total, 77 women and 82 men from WWC and 75 women and 85 men from PAWC were included in this study (Table 1 and Table 2). A total of 159 lifts from WWC and 160 lifts from PAWC were analyzed. 

The International Weightlifting Federation changed the official women’s weight categories in 2016. Thus, the women’s 90 kg category from PAWC was excluded from comparative analysis, and the women’s +75 category from WWC and +90 category from PAWC were considered equivalent during comparative analysis.

The ethics committee of East Tennessee State University determined this study to not be human subjects research. Therefore, subjects were not required to provide informed consent for inclusion in this study.

### 2.2. Data Collection

Lifts were recorded at both competitions using a GoPro HERO4 Black digital video camera (San Mateo, CA, USA) in 720p resolution (1280 × 720 pixels (px)) at 240 fps. Camera setup conformed to recommendations to minimize measurement error of sagittal plane barbell kinematics [23]. The camera was arranged on a tripod 15 m and 11 m from the left edge of the competition platform at WWC and PAWC, respectively, to avoid interference with competition proceedings. The camera lens was centered and leveled 0.71 m above the competition platform surface facing in line and parallel with the platform center for both competitions.

### 2.3. Video Analysis

Video analysis was conducted using Kinovea software (version 0.8.27, Kinovea open source project). The software’s working area was set to 400% zoom to improve accuracy of digitized marker placement and distance calibration. Kinovea’s automatic tracking feature was used to determine barbell displacement, recorded in px, by placing a digitized marker on the center of the visible end of the barbell prior to lift-off. Raw displacement data were exported to a spreadsheet for subsequent analysis using a custom Labview program. Raw data were converted from px to cm using a respective scaling coefficient (WWC: 0.71 cm∙px^−1^; PAWC: 0.67 cm∙px^−1^) and smoothed using a 20-point moving average. Outcome variables were determined from the converted, smoothed data.

#### Calibration Variability and Inter-Rater Reliability

The authors sought to determine the reasonableness of using a uniform scaling coefficient for each competition applied to each lift from that competition versus individually calibrating each video file to improve the time-efficiency of such analysis for coaches and researchers. Thus, 20 lifts from each competition were analyzed using both a uniform scaling coefficient and their individual scaling coefficient using the following procedures. Ten lifts each for both women and men with at least one lift from each weight category were randomly selected from each competition. Kinovea’s line tool was used to draw a line on the image along the vertical diameter of the largest plate (45 cm) nearest to the camera prior to lift-off. The length of the line was recorded in px and converted to a scaling factor (cm:px) for each file. The mean scaling factor for each set of 20 lifts was also determined. Outcome variables defined in Section 2.4 were determined for each file using both the original scaling factor corresponding to each file and the respective mean scaling factor.

The mean ± SD of calibration factors was 0.71 ± 0.007 cm∙px^−1^ for WWC and 0.67 ± 0.008 cm∙px^−1^ for PAWC (95% confidence intervals: 0.70 to 0.71 cm∙px^−1^ and 0.67 to 0.68 cm∙px^−1^, respectively). The 95% confidence intervals for % coefficient of variation for each set of individual calibration scales was 0.6 to 1.3% for WWC and 0.8 to 1.6% for PAWC.

Repeated measures ANOVAs for each outcome variable revealed no statistical differences between methods (F_(1,19)_ = 0.00040 to 0.23; *p* = 0.6 to > 0.9 and F_(1,19)_ = 0.0024 to 0.14; *p* = 0.7 to > 0.9 for WWC and PAWC, respectively). 95% confidence intervals for Pearson’s correlation coefficients for pairs of outcome variables determined from both methods were r = 0.988 to 0.999 and r = 0.982 to 0.999 for WWC and PAWC respectively (*p* < 0.001). Similar 95% confidence intervals for standard error of measurement between calibration methods for each variable were observed for WWC and PAWC (V_max_: −0.01 to 0.04 m∙s^−1^ vs. −0.01 to 0.05 m∙s^−1^; Y_max_: −0.7 to 2.1 cm vs. −0.8 to 2.5 cm; Y_catch_: −0.6 to 1.8 cm vs. −0.7 to 2.1 cm; Y_drop_: −0.09 to 0.3 cm vs. −0.1 to 0.4 cm; X_1_: −0.03 to 0.09 cm vs. −0.05 to 0.2 cm; X_2_: −0.05 to 0.2 cm vs. −0.05 to 0.1 cm; X_loop_: −0.04 to 0.1 cm vs. −0.03 to 0.1 cm; X_net_: −0.07 to 0.2 cm vs. −0.1 to 0.3 cm, respectively). 95% confidence intervals for ICC reliability coefficients for the determination of all outcome variables between both methods were 0.977 to 0.999 and 0.969 to 0.999 for WWC and PAWC, respectively, based on a single measure, absolute agreement, 2-way random effects model (*p* < 0.001). Thus, the likely amount of error between the two methods was deemed negligible and the respective standard scaling coefficients were applied to the sets of lifts from each competition to improve the time-efficiency of the analysis.

### 2.4. Outcome Variables

**Figure 1:**
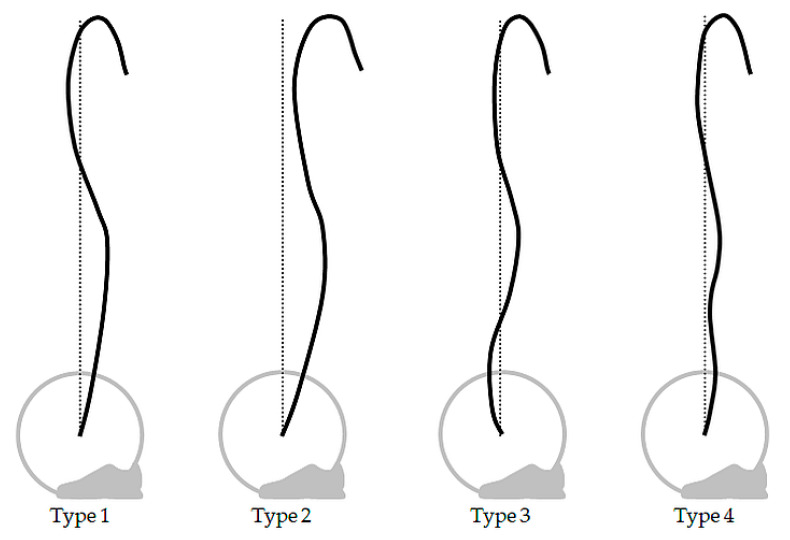
Barbell trajectory types determined by pattern of horizontal displacement and crossing of vertical reference line. Figure redrawn and adapted from Vorobyev [5] and Hiskia [4].

Barbell trajectory type was determined based on the classification scheme devised by Vorobyev [5], including the type 4 trajectory first introduced by Hiskia [4] (Figure 1). The authors of the present study considered the large number of lifts included in this study as an opportunity to explore the prevalence of the type 4 trajectory, which, to the authors’ knowledge, has not been quantified in the literature since its first identification by Hiskia [4]. Barbell trajectory was classified based on the pattern of horizontal barbell displacement relative to the lifter and crossing of a vertical reference line drawn intersecting the center of the barbell immediately prior to lift-off. The type 1 trajectory exhibits a ‘toward-away-toward’ pattern, crossing the vertical reference line during the ‘away’ phase and may or may not cross during the final ‘toward’ phase. The type 2 trajectory also consists of a ‘toward-away-toward’ pattern but does not cross the vertical reference line at any instant during the lift. The type 3 trajectory shows an ‘away-toward-away-toward’ pattern. The type 4 pattern may begin with a ‘toward’ phase, as in the type 1 or 2 trajectories, or an ‘away-toward’ phase, as in the type 3 trajectory. The defining feature of the type 4 trajectory is an interceding ‘away-toward’ phase between the first ‘toward’ phase and the final ‘away-toward’ phase. Only the type 1 trajectory necessarily crosses the vertical reference line.

**Figure 2:**
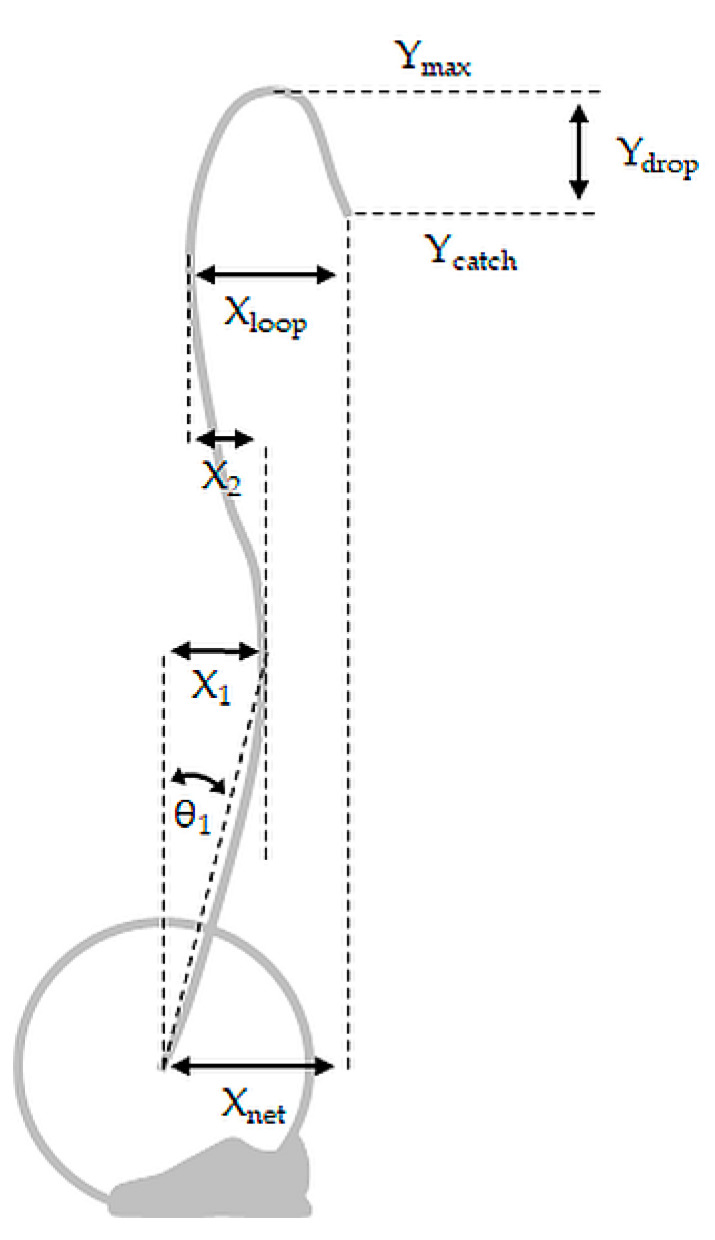
Barbell kinematic variables of displacement. Y_max_, maximum height; Y_catch_, height at catch; Y_drop_, difference between Y_max_ and Y_catch_; θ_1_, angle relative to vertical reference line from start position to X_1_; X_1_, net horizontal displacement from start position to most rearward position during first phase of displacement toward the lifter; X_2_, horizontal distance from X_1_ to most anterior position between X_1_ and Y_max_; X_loop_, horizontal distance from X_2_ to Y_catch_; X_net_, net horizontal displacement from start position to Y_catch_. Modified and adapted from Stone et al. [17].

Barbell kinematic variables were modified from Stone et al. [17] and included peak vertical velocity (V_max_), maximum barbell height (Y_max_), height at catch (Y_catch_), difference between Y_max_ and Y_catch_ (Y_drop_), ratio of Y_catch_ divided by Y_max_ (Catch_rel_), angle relative to vertical reference line from start position to position at X_1_ (θ_1_), net horizontal displacement from start position to most rearward position during first phase of displacement toward the lifter (X_1_), horizontal distance from X_1_ to most anterior position between X_1_ and Y_max_ (X_2_), horizontal distance from position at X_2_ to position at catch (X_loop_), net horizontal displacement from start position to Y_catch_ (X_net_) (Figure 2). For determination of Y_drop_ and X_loop_, the catch was defined as the first instance after the phase of negative vertical velocity following Y_max_ that the barbell reached a vertical velocity of 0 m∙s^−1^.

### 2.5. Statistical Analysis

The count and relative frequency of each barbell trajectory type were categorized by competition, sex, weight category, and continent. Descriptive statistics of each kinematic variable from all A session lifts separated by competition, sex, and weight category were calculated.

Separate omnibus 2 × 3 between-subject ANOVAs for women and men were conducted to compare the main effects of competition (WWC, PAWC) and placement (1, 2, 3) and the interaction effect of competition and placement on each of the kinematic variables from the top-three women and men finishers from both WWC and PAWC. Assumptions of normality and homoscedasticity were assessed using the Shapiro–Wilk test and Levene’s test, respectively. In cases where assumptions of normality or homoscedasticity were violated, a robust ANOVA procedure was conducted [24]. Critical alpha was set at α = 0.05. Cohen’s d effect size was calculated to evaluate the magnitude of all cell and marginal mean differences between groups.

## 3. Results

### 3.1. Descriptive Analysis

#### 3.1.1. Barbell Trajectory

Relative frequencies of each trajectory type were similar between WWC and PAWC. Top-three finishers at WWC and PAWC exhibited some differences in relative frequencies of trajectory types compared to A session lifters within and between competitions (Table 3; Supplementary Tables S1–S3).

The type 3 trajectory was the most prevalent type among all A session lifters at both WWC and PAWC (53% and 59%, respectively), with heavier men’s categories exhibiting a greater relative frequency than other categories. The type 3 trajectory was also the most common type among the top-three finishers at both competitions (43% and 49%, respectively); although, it was less prevalent among women compared to men at WWC (30% vs. 54%) and PAWC (43% vs. 54%). The type 2 trajectory was exhibited by ~30% of women and men at both WWC and PAWC. The type 1 trajectory accounted for 13% and 8% of lifts at WWC and PAWC, respectively. The type 4 trajectory occurred least frequently at both WWC and PAWC (6% and 3%, respectively).

A greater proportion of women’s top-three finishers at both WWC and PAWC exhibited the type 2 trajectory compared to their A session counterparts (50% vs. 29% and 39% vs. 29%, respectively). The top-three finishers in the men’s 105 and +105 categories at both WWC and PAWC exhibited the type 3 trajectory exclusively.

European top-three finishers from WWC most frequently exhibited the type 3 trajectory (57%), while Asian top-three finishers from WWC most frequently exhibited the type 2 trajectory (43%). There were no top-three finishers from North America, South America, or Africa at WWC at the time of competition. The type 4 trajectory accounted for 7% of the lifts among the top-three finishers at WWC.

South American top-three finishers at PAWC most commonly exhibited the type 3 trajectory (55%) followed by the type 2 trajectory (38%). North American top-three finishers at PAWC had an equal distribution of type 2 and 3 trajectories (39% each) with the remainder being the type 1 trajectory (22%). No top-three finishers at PAWC exhibited the type 4 trajectory.

#### 3.1.2. Kinematic Variables

Overall, the direction of the cell mean difference of most kinematic variables from WWC and PAWC was inconsistent over weight categories for both sexes (Table 4; Supplementary Table S4). Heavier lifters tended to exhibit greater V_max_, Y_max_, and Y_catch_, which is partly due to heavier lifters tending to also be taller [2,4,10,12]. Despite differences in Y_max_ and Y_catch_, Y_drop_ did not exhibit an increasing or decreasing trend based on weight category for either sex. Heavier lifters also tended to exhibit greater X_2_, which is likely partly attributable to differences in anthropometric variables among weight categories [12]. There were no discernible trends in barbell kinematics within weight categories among the top-three finishers for either sex at WWC or PAWC (Supplementary Tables S5–S8).

### 3.2. Comparative Analysis

#### 3.2.1. Women

Y_catch_, θ_1_, and X_1_ from PAWC did not meet the assumption of normality (*p* = 0.01, < 0.001, and 0.005, respectively; all other *p* = 0.1 to > 0.9). θ_1_ also did not satisfy the assumption of homoscedasticity (*p* = 0.002; all other *p* = 0.1 to > 0.9). A statistical main effect of competition was found only for Y_max_ (η^2^ = 0.106; F_(1,34)_ = 4.2; *p* = 0.049). Y_max_ mean ± SD was 0.96 ± 0.09 m for WWC and 1.02 ± 0.08 m for PAWC (Cohen’s d 95% confidence interval = −1.3 to −0.02). No statistical main or interaction effects were found for any other variable (*p* = 0.2 to > 0.9) (Supplementary Table S9). Magnitude and directionality of effect sizes varied considerably among variables (Cohen’s d = −0.87 to 0.77) (Supplementary Figure S1).

#### 3.2.2. Men

There was no variable that violated assumptions of normality or homoscedasticity (*p* = 0.1 to > 0.9). A statistical main effect of competition was found for X_2_ (η^2^ = 0.17; F_(1,42)_ = 8.8; *p* = 0.005), X_loop_ (η^2^ = 0.10; F_(1,42)_ = 5.6; *p* = 0.02), and X_net_ (η^2^ = 0.13; F_(1,42)_ = 6.5; *p* = 0.01). Mean ± SD for X_2_ was 0.06 ± 0.02 m for WWC and 0.04 ± 0.03 m for PAWC (Cohen’s d 95% confidence interval = 0.24 to 1.48). Mean ± SD for X_loop_ was 0.09 ± 0.04 m for WWC and 0.12 ± 0.05 m for PAWC (Cohen’s d 95% confidence interval = −1.3 to −0.06). Mean ± SD for X_net_ was 0.08 ± 0.08 for WWC and 0.14 ± 0.08 m for PAWC (Cohen’s d 95% confidence interval = −1.3 to −0.13). No statistical main or interaction effects were present for any other variable (*p* = 0.1 to > 0.9) (Supplementary Table S10). Effect sizes showed considerable variation with no clear pattern within or across factor levels (Cohen’s d = −1.06 to 0.68) (Supplementary Figure S2).

## 4. Discussion

The purpose of this descriptive study was to update the scientific knowledge of snatch technique of top international weightlifters. This study involved (1) descriptive analysis of barbell trajectory and kinematics of the snatch lift for A session lifters at two major international competitions and (2) comparative analysis of barbell kinematics between top-three performers in the snatch lift from each meet.

The relative frequencies of both type 2 and 3 trajectories for women pooled from WWC and PAWC were similar to those observed for women at the 1993 and 1994 World and European Weightlifting Championships [4]. Pooled data show that women at WWC and PAWC exhibited the type 1 trajectory less frequently than the women included in the report by Hiskia (13% vs. 22%) [4]. Antoniuk et al. observed from 137 women competing at several World and European Weightlifting Championships that the type 2 trajectory was most common [25]. A subsequent report by this group presented data from a total of 304 attempts by 140 women presumably from the same competitions reported in their first study that the type 1 trajectory was most common with the remainder sharing an equal distribution of type 2 and 3 trajectories [15]. Both studies reported a greater prevalence of the type 3 trajectory among the +75 category [15,25]. Pooled data for men at WWC and PAWC show similar relative frequency of the type 1 trajectory, lower relative frequency for the type 2 trajectory (~14%), and higher relative frequency for the type 3 trajectory (~9%) compared to men at the 1993 and 1994 World and European Weightlifting Championships [4]. To the authors’ knowledge, the present study is the first to quantify the prevalence of the type 4 trajectory at any competitive level since Hiskia [4] first reported this trajectory type in 1997; it is thus unknown how reclassification of data from prior studies to include the type 4 trajectory would affect comparison to the results of the present study.

The present results corroborate the findings of Akkuş [9], who observed that female world champions at the 2010 World Weightlifting Championship exhibited a variety of trajectory types. Musser et al. [12] found that no women snatch medalists at the 2009 Pan-American Weightlifting Championship exhibited the type 1 trajectory. However, the results of the present study demonstrate that top-three women at the Pan-American championship level can also likely exhibit a variety of trajectory types including the type 1 trajectory. The differences in relative frequencies of trajectory types between this study and previous reports [4,12,15,25] are likely due to different athlete pools, and it is unclear whether the success of certain trajectory types are exclusive to or more likely to occur in particular weight categories. However, it is likely from these differences that the relative frequencies of trajectory types observed among women at different major international competitions do vary and that high placement is not exclusive to any existing trajectory type. Although, some trajectory types may potentially be more common among the top performers in some weight categories partly due to anthropometry [10,12] or other factors.

Although Baumann et al. [6] did not report counts or relative frequencies of barbell trajectory type, they did indicate that ‘nearly all’ men in a sample of A session lifters at the 1985 World Weightlifting Championship exhibited the type 2 trajectory, with the remainder presumably exhibiting the type 1 trajectory since the authors did not report any occurrences of the type 3 trajectory. Limited data from Garhammer [26,27] do indicate the presence of the type 3 trajectory for the snatch and the clean among women and men world and Olympic champions during the 1980s. However, the type 3 trajectory does not appear to have been common among the top international lifters during that period [6,26]. In fact, based on serial observations, Garhammer [26] suggested that technique assessed by barbell trajectory had not changed from the mid-1970s to the late-1980s. Subsequently, Hiskia [4] found the type 3 trajectory to be the most common among both women and men in the A sessions of the 1993 and 1994 World and European Weightlifting Championships. Data from Akkuş [9] of all women snatch gold medalists and Harbili [11] of women and men in the A session of their respective 69 kg categories at the 2010 World Weightlifting Championship indicate increased prevalence of the type 3 trajectory among top international weightlifters at that competition. Collectively, these data seem to indicate that the relative frequencies of barbell trajectory types likely vary between and within international competitions. The available evidence indicates a likely shift in technique based on barbell trajectory among international weightlifters since as early as the late-1980s. Inclusion of the type 4 trajectory in future analyses may also help to reveal any changes in the prevalence of the type 4 trajectory over time.

The observed differences in the prevalence of trajectory types among the top international weightlifters may reflect changes in coaching philosophy, teaching methods, and/or training methods to accommodate what have previously been considered ‘suboptimal’ technique (i.e., type 2 and 3 trajectories) [5,9,26]. Furthermore, the differences observed between women in this study and women in previous investigations may partially be due to the selective pressure of elite competition. Given the longer history of men’s weightlifting, such selective pressure is less likely to be a current factor for men. Kinematic differences due to body size and anthropometry [4,10,12] may also partly explain observed differences for both sexes due to periodic changes in weight categories. Serial investigations can help to delineate any apparent trends across extant and future analyses. Furthermore, continued study of the relative frequency of trajectory types over a range of competitive levels and subgroups among them can help to identify which trajectory type(s), if any, is most characteristic of a given group.

The observations in this and recent studies [9,11] of high placing individuals at the World championship level who exhibit the type 3 trajectory is noteworthy. The type 1 trajectory was initially considered to be most favorable [5,26]. Some authors have more recently suggested the type 2 trajectory to be advantageous [17]. However, the type 3 trajectory is generally regarded to be non-beneficial and potentially detrimental [5,17] based on several biomechanical and theoretical bases. Garhammer and Taylor [28] found that anterior barbell displacement at lift-off, such as occurs with the type 3 trajectory, results in a forward shift of the lifter’s center of pressure, or balance point. Such barbell displacement also increases moment arm length between the barbell center of mass and joint centers, thereby increasing joint moments and muscular forces required to lift the barbell [6]. Furthermore, anterior displacement during the first pull can increase mechanical work and decrease lift efficiency [29,30]. Thus, the prevalence of the type 3 trajectory among the top-three finishers—especially among the men’s 105 and +105 categories, who lifted the heaviest loads—at both WWC and PAWC emphasize the importance of high levels of absolute strength, possibly to overcome apparent technical deficiencies [17]. Greater lower limb length may also partly explain the increased prevalence of the type 3 trajectory among heavier categories [12], and anthropometric variables may partly explain differences in the prevalence of trajectory types more generally [12,31]. Nonetheless, numerous observations of the gamut of trajectory types among A session and top-three international weightlifters somewhat challenges the notion that barbell trajectory type is a useful criterion of effective weightlifting technique at this level.

Few statistical effects were observed in this study. The observed main effect of competition for Y_max_ among the top-three women in this study could be due to differences in stature [2,6,10,32], skill [7,8], or load lifted [32]. However, the lack of accompanying statistical or clear effect size differences for Y_drop_, which has also been suggested to depend on skill [7,8], and observations of weightlifters who lifted heavier loads to greater absolute and relative vertical displacements compared to lower caliber athletes in the same weight category [8] suggest stature to be a more likely explanation of Y_max_ differences observed in this study. Top-three men at WWC, who lifted the heaviest loads of any group in this study, exhibited greater X_2_ and less X_net_ and X_loop_ than top-three men at PAWC. It is unlikely that greater X_2_ itself is beneficial for performance, as greater X_2_ would increase the overall work and energy required to complete the lift [6,33,34,35] and may increase instability during the catch [6]. These effects could be compounded by a potential subsequent increase in X_loop_, which is likely to be less during successful attempts [17]. Greater X_2_ among individuals of greater weightlifting ability may be consequent to the larger forces and accelerations associated with lifting heavier loads [3,6,8,14,36]. As such, it is not recommended that individuals attempt to deliberately increase X_2_ such as by ‘hipping’ or swinging the bar away during the second pull [17,37]. The reduced X_loop_ and X_net_ among the top-three men at WWC likely indicate that they jumped backward less than top-three men at PAWC. These results support the findings of Stone et al. that, while net rearward displacement is generally not detrimental or disadvantageous, smaller relative X_loop_ and X_net_ are likely associated with greater weightlifting success and ability [17]. Several authors have suggested that the direction of force application is important especially during the second pull [17,37,38]. The observations of reduced X_loop_ and X_net_ among stronger weightlifters possibly reflect these individuals’ ability to produce greater vertical force and acceleration [3,6,8,35,39]. Stronger individuals are also more likely to produce faster rates of force development [40], which may improve their ability to counteract greater X_2_ through a more immediate reversal of anterior horizontal barbell acceleration during the second pull and turnover phase thereby reducing X_loop_ and X_net_ [37,38]. One plausible explanation incorporating these results is that greater strength may improve energy flow, force application, and vertical acceleration to favorably influence horizontal barbell kinematics that affect weightlifting performance and ability.

The lifters in this study generally lifted heavier loads compared to lifters in the same or similar weight categories in previous studies [6,9,11,12,35] while exhibiting no clear differences in technique, suggesting that other factors may help to explain differences in weightlifting ability. Studies that have identified kinematic and kinetic differences based on skill or sex have consistently identified factors that partly depend on strength [8,9,11,36]. For example, greater strength improves the ability to perform stretch-shortening cycle tasks [41,42,43], such as occurs during the transition phase of the weightlifting pull. Several studies have also made direct comparisons between different groups of weightlifters (e.g., women vs. men [44], adolescent vs. adult [36], district vs. national/international [14]) and found overall similarity in kinematic and kinetic structure. However, when considering differences in weightlifting ability, there are notable differences in kinetic variables such as maximum ground reaction force [3,14,45], rate of force development [14,46], and absolute and relative joint and whole body power [30,36,47,48,49], which are all dependent on maximum strength. Indeed, while direct causal evidence is sparse, there exists strong relationships between measures of maximum strength and weightlifting ability among weightlifters of a variety of competitive levels. For example, Lucero et al. reported Pearson’s r = 0.91 to 0.94 for the relationships between self-reported back and front squat one-repetition maximums (1RMs) and snatch and clean 1RMs among male competitive weightlifters in the United States [50]. Stone et al. reported Pearson’s r = 0.79 to 0.95 for the relationships between back squat 1RM and snatch and clean 1RMs and Pearson’s r = 0.83 to 0.84 for the relationships between isometric mid-thigh pull peak force and snatch and clean 1RMs in national and international level junior and senior weightlifters from the United States [51]. Joffe and Tallant similarly observed Pearson’s r = 0.83 for the relationship between isometric mid-thigh pull peak force and the snatch among British international women weightlifters [52]. Collectively, these data provide some evidence toward the notion that strength is an important determinant of weightlifting ability.

The observed variety of barbell trajectory types and overall lack of pattern among individual or clusters of kinematic variables in this study suggest no standard ‘technique profile’ is requisite for high achievement in weightlifting. In fact, several investigations have had limited success differentiating kinematic profiles of successful versus unsuccessful weightlifting attempts [37,38]. However, such findings should not be interpreted to suggest that technique is not an important determinant of weightlifting success at any level. Rather, these findings more likely reflect the fact that the weightlifting movements are influenced by multiple degrees of freedom [17,53,54,55], and the varied results of this study may reflect individualized solutions to the degrees of freedom problem in weightlifting. The results of this study thus suggest the possibility of a variety of effective individualized technique profiles for weightlifting performance and ability.

It is purported that individual weightlifting technique stabilizes after only a few months of training [1,56]. However, the amount of intra-individual variation of lifter or barbell kinematics and kinetics during maximal attempts under competition conditions is not well defined. The results presented in two studies by Antoniuk et al., which analyzed different sets of snatch attempts from the same pool of athletes and competitions, substantiate that an individual can exhibit different barbell trajectories between attempts [15,25]. Five athletes in the present study competed in both WWC and PAWC. Incidentally, each athlete exhibited the same barbell trajectory for their respective lifts analyzed in this study. Nonetheless, the reliability of individual technique profiles remains unclear. These uncertainties notwithstanding, individual technique profiles consisting of any or some technical or biomechanical parameters may potentially be useful for evaluating, monitoring, or predicting weightlifting performance and ability.

There are a variety of laboratory- and field-based technologies available to conduct weightlifting technique analysis, and both researchers and coaches should explore the development of individual technique profiles. The methods of this study provide support for the implementation of video analysis for determining barbell kinematics and developing technique profiles. The total time to analyze a single video file including calibration during this study was less than two minutes. Use of Kinovea’s native graphics and analysis functions could reduce this time to 15 to 30 s.

Additionally, Carson et al. reported a case study that demonstrates the use of long-term monitoring of lifter kinematics during an intervention intended to change weightlifting technique [57]. This case study and the anecdotal experiences of athletes and coaches highlight the complexity and difficulty of instilling changes to technique that are robust enough to persist during attempts at high relative intensities [57]. Thus, the relatively short latency of weightlifting technique stabilization and the associated challenges of technique correction underscore the importance of establishing sound technique during the earliest stages of a weightlifter’s career and highlight the practical importance of strength development in the long-term improvement of weightlifting performance.

Instructional and coaching methods should generally be guided by principles and tenets from the fields of biomechanics, motor learning, and physiology. While the available evidence has not identified universal optimal technique, which is unlikely to exist, there are general guidelines for basic weightlifting technique apparent from the extensive body of scientific literature [2,3,4,5,6]. However, coaches should consider and make appropriate accommodations for individual differences that may manifest nuances or peculiarities in technique.

## 5. Conclusions

The methods used in this study demonstrate an inexpensive, time-efficient, and user-friendly method to conduct technique analysis. The error and reliability of the methods herein can be improved by optimizing camera setup and specifications [23,34,58], controlling conditions during data collection (e.g., barbell placement [59] and lighting [60]), and selecting appropriate data smoothing or filtering techniques [60,61,62]. Coaches may also elect to use a uniform calibration factor given consistent camera and platform setup to further improve time-efficiency of video analysis. Video analysis may aid coaches in determining barbell kinematics and developing technique profiles. Such technique profiles may be useful in the evaluation, monitoring, or prediction of weightlifting performance and ability. However, the individual reliability of weightlifting technique at any level requires further determination.

A variety of barbell trajectories were observed with differences based on competition, sex, weight category, and ranking. The results of this study indicate that weightlifting success can be achieved with a variety of technique profiles. While practically relevant, barbell trajectory or any of the examined kinematic variables alone is unlikely to reliably indicate weightlifting ability at this level. Therefore, coaches may consider evaluating weightlifting technique within a more general framework.

A lack of consistent statistical or effect size differences among the top-three women or top-three men suggests that other factors, such as strength, may help to explain differences in weightlifting performance and ability. Furthermore, the short latency of technique stabilization [1,56] and subsequent difficulty of creating robust changes in technique [57] highlight the practical importance of strength in the long-term improvement of weightlifting performance and ability, with one study observing that strength alone accounted for 42.5% of the variance in the change of weightlifting performance [52]. Thus, once an individual’s technique is established, weightlifting training should primarily emphasize the development of strength and other related physical characteristics using a periodized approach [63,64] while incorporating complementary methods to refine technique [57]. Video analysis is a viable method for coaches to evaluate, monitor, and refine weightlifting technique.

## Figures and Tables

**Table 1:** Athlete characteristics for 2015 World Weightlifting Championship.

Category	*n*	Age (y)	BW (kg)
Women			
48	11	25.5 ± 5.3	47.65 ± 0.23
53	12	23.3 ± 3.6	52.53 ± 0.71
58	11	26.3 ± 4.9	57.49 ± 0.51
63	12	23.3 ± 3.4	62.45 ± 0.59
69	10	23.3 ± 3.7	68.24 ± 0.81
75	10	25.3 ± 4.0	74.53 ± 0.40
+75	11	24.5 ± 2.6	114.06 ± 16.69
	77	24.5 ± 3.3	67.77 ± 21.64
Men			
56	7	24.1 ± 4.0	55.79 ± 0.13
62	11	25.4 ± 3.0	61.82 ± 0.14
69	11	24.4 ± 2.9	68.78 ± 0.15
77	11	24.7 ± 4.1	76.60 ± 0.30
85	10	24.2 ± 3.0	84.36 ± 0.38
94	10	25.0 ± 2.4	93.46 ± 0.48
105	10	24.9 ± 3.4	104.54 ± 0.45
+105	12	26.5 ± 3.7	148.74 ± 6.96
	82	25.0 ± 3.3	88.76 ± 29.02

Values are the mean ± SD; BW, bodyweight.

**Table 2:** Athlete characteristics for 2017 Pan-American Weightlifting Championship.

Category	*n*	Age (y)	BW (kg)
Women			
48	11	24.9 ± 4.8	47.67 ± 0.31
53	9	25.5 ± 6.0	52.35 ± 1.10
58	9	27.2 ± 5.2	57.44 ± 0.68
63	9	27.1 ± 5.1	62.70 ± 0.28
69	9	21.9 ± 3.3	67.89 ± 0.96
75	10	24.8 ± 4.3	74.58 ± 0.38
90	9	25.7 ± 6.4	85.58 ± 4.22
+90	9	24.4 ± 5.2	110.01 ± 18.24
	75	25.2 ± 5.1	69.25 ± 20.07
Men			
56	10	23.2 ± 6.4	55.78 ± 0.23
62	15	22.5 ± 3.1	61.40 ± 0.60
69	14	23.8 ± 4.1	68.31 ± 0.62
77	10	21.4 ± 3.3	76.54 ± 0.56
85	8	24.3 ± 5.6	84.42 ± 0.43
94	10	25.4 ± 4.3	92.29 ± 2.62
105	9	24.7 ± 2.0	103.88 ± 1.22
+105	9	25.8 ± 3.7	135.76 ± 18.17
	85	23.8 ± 4.2	81.83 ± 24.47

Values are the mean ± SD; BW, bodyweight.

**Table 3:** Distribution of barbell trajectory type of each athlete’s heaviest successful snatch attempt in A sessions at 2015 World Weightlifting Championship.

Category	Type 1	Type 2	Type 3	Type 4
Women				
48	-	7 (64)	4 (36)	-
53	1 (8)	1 (8)	8 (67)	2 (17)
58	1 (9)	5 (45)	5 (45)	-
63	2 (17)	3 (25)	7 (58)	-
69	1 (10)	4 (40)	5 (50)	-
75	4 (40)	1 (10)	5 (50)	-
+75	1 (9)	1 (9)	9 (82)	-
	10 (13)	22 (29)	43 (56)	2 (3)
Men				
56	2 (29)	3 (43)	1 (14)	1 (14)
62	3 (27)	6 (55)	1 (9)	1 (9)
69	1 (9)	3 (27)	5 (45)	2 (18)
77	1 (9)	5 (45)	4 (36)	1 (9)
85	2 (20)	1 (10)	7 (70)	-
94	-	2 (20)	8 (80)	-
105	1 (10)	-	8 (80)	1 (10)
+105	-	3 (25)	8 (67)	1 (8)
	10 (12)	23 (28)	42 (51)	7 (9)
Continent				
North America	1 (17)	2 (33)	3 (50)	-
South America	3 (25)	4 (33)	4 (33)	1 (8)
Asia	6 (9)	26 (39)	31 (46)	4 (6)
Europe	9 (14)	10 (15)	43 (65)	4 (6)
Africa	1 (13)	3 (38)	4 (50)	-
Grand Total	20 (13)	45 (28)	85 (53)	9 (6)

Values are count (% relative frequency).

**Table 4:** Load and kinematic variables for heaviest successful snatch attempt for A session lifters at the 2015 World Weightlifting Championship.

Category	Load	V_max_	Y_max_	Y_catch_	Y_drop_	Catch_rel_	θ_1_	X_1_	X_2_	X_loop_	X_net_
	(kg)	(m∙s^−1^)	(m)	(m)	(m)	(%)	(°)	(m)	(m)	(m)	(m)
Women											
48	82 ± 4	1.82 ± 0.10	0.89 ± 0.05	0.73 ± 0.04	0.16 ± 0.04	82 ± 4	7 ± 3	0.05 ± 0.02	0.05 ± 0.02	0.09 ± 0.04	0.09 ± 0.07
53	90 ± 6	1.81 ± 0.11	0.93 ± 0.05	0.77 ± 0.06	0.16 ± 0.04	83 ± 4	5 ± 3	0.04 ± 0.02	0.05 ± 0.02	0.10 ± 0.04	0.09 ± 0.06
58	100 ± 7	1.84 ± 0.13	0.95 ± 0.04	0.79 ± 0.05	0.17 ± 0.04	83 ± 4	7 ± 4	0.06 ± 0.03	0.05 ± 0.02	0.11 ± 0.04	0.12 ± 0.09
63	104 ± 7	1.90 ± 0.11	0.97 ± 0.06	0.81 ± 0.05	0.16 ± 0.03	83 ± 3	6 ± 4	0.06 ± 0.03	0.06 ± 0.03	0.10 ± 0.06	0.11 ± 0.11
69	112 ± 6	1.78 ± 0.11	0.98 ± 0.06	0.82 ± 0.06	0.16 ± 0.04	84 ± 4	6 ± 3	0.06 ± 0.03	0.06 ± 0.02	0.11 ± 0.04	0.12 ± 0.08
75	114 ± 9	1.88 ± 0.10	1.02 ± 0.05	0.86 ± 0.06	0.16 ± 0.03	84 ± 3	5 ± 2	0.05 ± 0.02	0.08 ± 0.03	0.09 ± 0.05	0.06 ± 0.10
+75	126 ± 12	1.99 ± 0.11	1.10 ± 0.06	0.96 ± 0.06	0.15 ± 0.03	87 ± 2	7 ± 2	0.07 ± 0.02	0.07 ± 0.02	0.15 ± 0.05	0.15 ± 0.08
	104 ± 16	1.86 ± 0.13	0.98 ± 0.08	0.82 ± 0.09	0.16 ± 0.04	84 ± 4	6 ± 3	0.06 ± 0.03	0.06 ± 0.03	0.11 ± 0.05	0.10 ± 0.09
Men											
56	126 ± 9	1.79 ± 0.11	0.92 ± 0.07	0.77 ± 0.06	0.15 ± 0.03	84 ± 3	10 ± 5	0.06 ± 0.03	0.05 ± 0.02	0.08 ± 0.03	0.09 ± 0.05
62	138 ± 7	1.77 ± 0.07	0.92 ± 0.01	0.77 ± 0.04	0.15 ± 0.04	84 ± 4	7 ± 4	0.06 ± 0.04	0.05 ± 0.03	0.09 ± 0.07	0.10 ± 0.14
69	149 ± 8	1.73 ± 0.09	0.95 ± 0.04	0.79 ± 0.06	0.16 ± 0.04	83 ± 4	6 ± 2	0.05 ± 0.02	0.05 ± 0.02	0.10 ± 0.03	0.11 ± 0.06
77	162 ± 7	1.82 ± 0.09	0.99 ± 0.04	0.86 ± 0.04	0.13 ± 0.04	87 ± 4	7 ± 3	0.06 ± 0.02	0.05 ± 0.02	0.09 ± 0.04	0.10 ± 0.07
85	168 ± 7	1.83 ± 0.12	1.02 ± 0.05	0.89 ± 0.03	0.13 ± 0.04	87 ± 4	5 ± 1	0.04 ± 0.01	0.06 ± 0.02	0.09 ± 0.04	0.07 ± 0.07
94	176 ± 4	1.84 ± 0.10	1.07 ± 0.03	0.90 ± 0.06	0.17 ± 0.04	84 ± 4	5 ± 3	0.05 ± 0.04	0.07 ± 0.02	0.09 ± 0.03	0.08 ± 0.09
105	182 ± 6	1.88 ± 0.08	1.10 ± 0.03	0.93 ± 0.05	0.17 ± 0.03	84 ± 3	5 ± 2	0.05 ± 0.02	0.08 ± 0.02	0.11 ± 0.03	0.09 ± 0.05
+105	196 ± 8	1.88 ± 0.14	1.17 ± 0.08	1.02 ± 0.07	0.15 ± 0.04	87 ± 3	6 ± 3	0.07 ± 0.03	0.08 ± 0.02	0.13 ± 0.05	0.12 ± 0.09
	164 ± 22	1.82 ± 0.11	1.02 ± 0.10	0.87 ± 0.10	0.15 ± 0.04	85 ± 4	6 ± 3	0.06 ± 0.03	0.06 ± 0.03	0.10 ± 0.05	0.10 ± 0.08

Values are the mean ± SD.

## Supplementary Materials

The following are available online at https://www.mdpi.com/2075-4663/8/9/118/s1, Figures S1 and S2; Supplementary Tables S1–S10.
