# Supplementary material for: Survey of Barbell Trajectory and Kinematics of the Snatch Lift from the 2015 World and 2017 Pan-American Weightlifting Championships
Source: Sports (Basel). 2020 Aug 25;8(9):118. doi: 10.3390/sports8090118 (PMC7552656; doi:10.3390/sports8090118)
Supplement: Supplementary file 1 [file sports-08-00118-s001.zip › Figure S2.pdf]

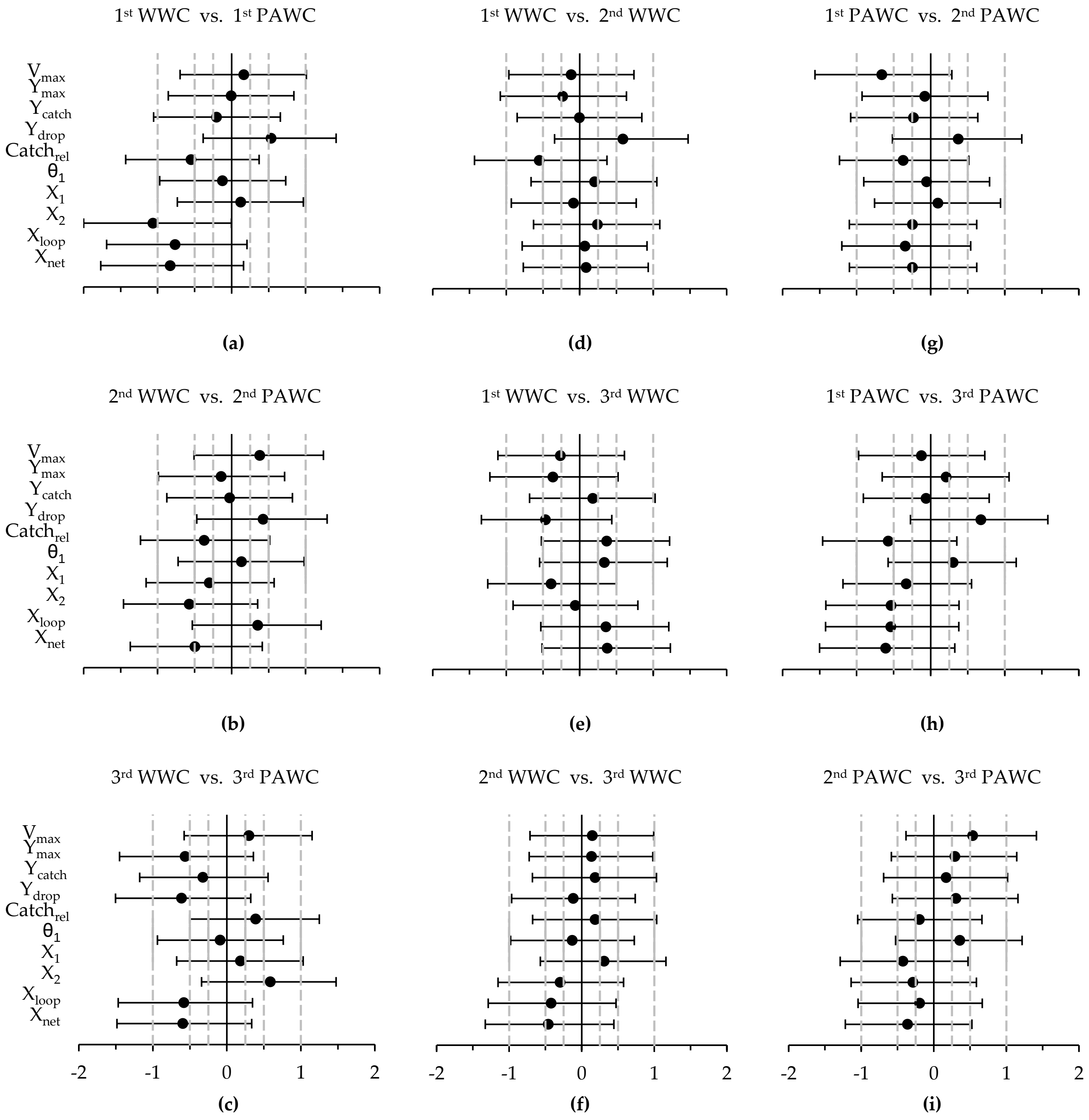

Figure S2. Cohen's d effect size with 95% confidence interval for main effects of competition **(a-c)** and placement **(d-i)** among top-three men: **(a)** 1st place at WWC vs. 1st place at PAWC; **(b)** 2nd place at WWC vs. 2nd place at PAWC; **(c)** 3rd place at WWC vs. 3rd place at PAWC; **(d)** 1st place at WWC vs. 2nd place at WWC; **(e)** 1st place at WWC vs. 3rd place at WWC; **(f)** 2nd place at WWC vs. 3rd place at WWC; **(g)** 1st place at PAWC vs. 2nd place at PAWC; **(h)** 1st place at PAWC vs. 3rd place at PAWC; **(i)** 2nd place at PAWC vs. 3rd place at PAWC; WWC, 2015 World Weightlifting Championship; PAWC, 2017 Pan-American Weightlifting Championship.
