# Supplementary material for: Survey of Barbell Trajectory and Kinematics of the Snatch Lift from the 2015 World and 2017 Pan-American Weightlifting Championships
Source: Sports (Basel). 2020 Aug 25;8(9):118. doi: 10.3390/sports8090118 (PMC7552656; doi:10.3390/sports8090118)
Supplement: Supplementary file 1 [file sports-08-00118-s001.zip › Supplementary Table S1.pdf]

Table S1. Distribution of barbell trajectory type of each athlete's heaviest successful snatch attempt in A sessions at 2017 Pan-American Weightlifting Championship.

**Category**	**Type 1**	**Type 2**	**Type 3**	**Type 4**
Women				
48	2 (18)	6 (55)	2 (18)	1 (9)
53	-	6 (67)	3 (33)	-
58	2 (22)	2 (22)	5 (56)	-
63	1 (11)	3 (33)	5 (56)	-
69	1 (11)	1 (11)	6 (67)	1 (11)
75	-	2 (20)	8 (80)	-
90	-	1 (11)	8 (89)	-
+90	3 (33)	1 (11)	4 (44)	1 (11)
	9 (12)	22 (29)	41 (55)	3 (4)
Men				
56	1 (10)	2 (20)	7 (70)	-
62	1 (7)	11 (73)	3 (20)	-
69	-	4 (29)	9 (64)	1 (7)
77	-	4 (40)	6 (60)	-
85	1 (13)	2 (25)	5 (63)	-
94	1 (10)	1 (10)	8 (80)	-
105	-	1 (11)	8 (89)	-
+105	-	-	8 (89)	1 (11)
	4 (5)	25 (29)	54 (64)	2 (2)
Continent				
North America	9 (11)	26 (32)	46 (56)	1 (1)
South America	4 (5)	21 (27)	49 (63)	4 (5)
Grand Total	13 (8)	47 (29)	95 (59)	5 (3)

Values are count (% relative frequency).
