# Supplementary material for: Survey of Barbell Trajectory and Kinematics of the Snatch Lift from the 2015 World and 2017 Pan-American Weightlifting Championships
Source: Sports (Basel). 2020 Aug 25;8(9):118. doi: 10.3390/sports8090118 (PMC7552656; doi:10.3390/sports8090118)
Supplement: Supplementary file 1 [file sports-08-00118-s001.zip › Supplementary Table S10.pdf]

Table S10. Results of omnibus 2x3 between-subjects ANOVAs for men.

	**Competition**			**Placement**			**Competition by placement**		
	**η^2**	**F**	**p**	**η^2**	**F**	**p**	**η^2**	**F**	**p**
V_{max}	0.00026	0.01	> 0.9	0.036	0.81	0.5	0.031	0.69	0.5
Y_{max}	0.020	0.87	0.4	0.0059	0.13	0.9	0.021	0.47	0.6
Y_{catch}	0.0097	0.45	0.5	0.010	0.24	0.8	0.086	2.0	0.1
Y_{drop}	0.010	0.45	0.5	0.0046	0.10	> 0.9	0.0079	0.17	0.8
$Catch_{rel}$	0.0017	0.08	0.8	0.0087	0.20	0.8	0.068	1.5	0.2
θ_1	0.00029	0.01	> 0.9	0.027	0.59	0.6	0.0049	0.11	> 0.9
X_1	0.015	0.66	0.4	0.042	0.94	0.4	0.0020	0.04	> 0.9
X_2	0.17	8.8	0.005	0.024	0.62	0.5	0.016	0.41	0.7
X_{loop}	0.10	5.1	0.03	0.045	1.1	0.3	0.0090	0.22	0.8
X_{net}	0.13	6.6	0.01	0.057	1.5	0.2	0.0060	0.16	0.9
