# Supplementary material for: Survey of Barbell Trajectory and Kinematics of the Snatch Lift from the 2015 World and 2017 Pan-American Weightlifting Championships
Source: Sports (Basel). 2020 Aug 25;8(9):118. doi: 10.3390/sports8090118 (PMC7552656; doi:10.3390/sports8090118)
Supplement: Supplementary file 1 [file sports-08-00118-s001.zip › Supplementary Table S2.pdf]

Table S2. Distribution of barbell trajectory type of each top-three finisher's heaviest successful snatch attempt at 2015 World Weightlifting Championship.

Category	Type 1	Type 2	Type 3	Type 4
Women				
48	-	2	1	-
53	-	-	1	2
58	-	2	1	-
63	-	2	1	-
69	1	2	-	-
75*	1	1	-	-
+75	-	1	2	-
	2 (10)	10 (50)	6 (30)	2 (10)
Men				
56	1	1	1	-
62	2	1	-	-
69	1	-	1	1
77	-	1	2	-
85	1	1	1	-
94	-	1	2	-
105	-	-	3	-
+105	-	-	3	-
	5 (21)	5 (21)	13 (54)	1 (4)
Continent				
North America	-	-	-	-
South America	-	-	-	-
Asia	4 (13)	13 (43)	11 (37)	2 (7)
Europe	3 (21)	2 (14)	8 (57)	1 (7)
Africa	-	-	-	-
Grand Total	7 (16)	15 (34)	19 (43)	3 (7)

Values are count (% relative frequency); * one lift not recorded due to hardware/software error.
