# Supplementary material for: Survey of Barbell Trajectory and Kinematics of the Snatch Lift from the 2015 World and 2017 Pan-American Weightlifting Championships
Source: Sports (Basel). 2020 Aug 25;8(9):118. doi: 10.3390/sports8090118 (PMC7552656; doi:10.3390/sports8090118)
Supplement: Supplementary file 1 [file sports-08-00118-s001.zip › Supplementary Table S3.pdf]

Table S3. Distribution of barbell trajectory type of each top-three finisher's heaviest successful snatch attempt at 2017 Pan-American Weightlifting Championship.

Category	Type 1	Type 2	Type 3	Type 4
Women				
48*	1	1	-	-
53	-	2	1	-
58	-	2	1	-
63	1	1	1	-
69	1	-	2	-
75	-	2	1	-
90	-	-	3	-
+90	1	1	1	-
	4 (17)	9 (39)	10 (43)	-
Men				
56	-	2	1	-
62	-	2	1	-
69	-	2	1	-
77	-	2	1	-
85	1	-	2	-
94	1	1	1	-
105	-	-	3	-
+105	-	-	3	-
	2 (8)	9 (38)	13 (54)	-
Continent				
North America	4 (22)	7 (39)	7 (39)	-
South America	2 (7)	11 (38)	16 (55)	-
Grand Total	6 (13)	18 (38)	23 (49)	-

Values are count (% relative frequency); * one lift not recorded due to hardware/software error.
