# Supplementary material for: Survey of Barbell Trajectory and Kinematics of the Snatch Lift from the 2015 World and 2017 Pan-American Weightlifting Championships
Source: Sports (Basel). 2020 Aug 25;8(9):118. doi: 10.3390/sports8090118 (PMC7552656; doi:10.3390/sports8090118)
Supplement: Supplementary file 1 [file sports-08-00118-s001.zip › Supplementary Table S4.pdf]

Table S4. Load and kinematic variables for heaviest successful snatch attempt for A session lifters at 2017 Pan-American Weightlifting Championship.

Category	Load (kg)	V_{max} (m·s^{-1})	Y_{max} (m)	Y_{catch} (m)	Y_{drop} (m)	$Catch_{rel}$ (%)	θ_1 (°)	X_1 (m)	X_2 (m)	X_{loop} (m)	X_{net} (m)
Women											
48	68 ± 10	1.78 ± 0.10	0.92 ± 0.03	0.77 ± 0.05	0.16 ± 0.03	83 ± 3	8 ± 2	0.06 ± 0.01	0.05 ± 0.02	0.09 ± 0.03	0.10 ± 0.04
53	83 ± 3	1.77 ± 0.09	0.93 ± 0.05	0.76 ± 0.06	0.17 ± 0.03	81 ± 4	8 ± 2	0.08 ± 0.02	0.04 ± 0.02	0.12 ± 0.04	0.16 ± 0.06
58	89 ± 5	1.89 ± 0.08	0.99 ± 0.02	0.81 ± 0.03	0.18 ± 0.04	82 ± 4	5 ± 2	0.05 ± 0.02	0.05 ± 0.01	0.10 ± 0.02	0.09 ± 0.04
63	89 ± 5	1.88 ± 0.08	1.00 ± 0.04	0.86 ± 0.05	0.14 ± 0.03	86 ± 3	7 ± 2	0.06 ± 0.01	0.05 ± 0.03	0.13 ± 0.06	0.14 ± 0.09
69	93 ± 9	1.94 ± 0.10	1.05 ± 0.04	0.86 ± 0.04	0.19 ± 0.04	82 ± 3	5 ± 3	0.05 ± 0.03	0.07 ± 0.03	0.10 ± 0.04	0.08 ± 0.10
75	99 ± 6	1.85 ± 0.06	1.05 ± 0.04	0.87 ± 0.07	0.17 ± 0.04	83 ± 4	0 ± 18	0.05 ± 0.03	0.07 ± 0.03	0.11 ± 0.04	0.09 ± 0.08
90	101 ± 13	1.83 ± 0.08	1.04 ± 0.03	0.89 ± 0.04	0.15 ± 0.04	86 ± 4	6 ± 5	0.06 ± 0.05	0.07 ± 0.02	0.12 ± 0.04	0.11 ± 0.10
+90	99 ± 22	1.99 ± 0.12	1.12 ± 0.07	0.98 ± 0.09	0.14 ± 0.04	87 ± 4	8 ± 4	0.08 ± 0.04	0.08 ± 0.03	0.13 ± 0.05	0.13 ± 0.10
	90 ± 15	1.86 ± 0.11	1.01 ± 0.07	0.85 ± 0.09	0.16 ± 0.04	84 ± 4	6 ± 7	0.06 ± 0.03	0.06 ± 0.03	0.11 ± 0.04	0.11 ± 0.08
Men											
56	102 ± 10	1.77 ± 0.11	0.95 ± 0.07	0.81 ± 0.05	0.14 ± 0.04	85 ± 3	6 ± 3	0.05 ± 0.03	0.04 ± 0.03	0.11 ± 0.04	0.11 ± 0.09
62	113 ± 11	1.84 ± 0.16	0.97 ± 0.06	0.82 ± 0.07	0.14 ± 0.04	85 ± 4	9 ± 3	0.08 ± 0.03	0.03 ± 0.02	0.12 ± 0.06	0.17 ± 0.10
69	120 ± 16	1.79 ± 0.10	0.99 ± 0.05	0.85 ± 0.05	0.14 ± 0.04	86 ± 4	7 ± 3	0.07 ± 0.04	0.06 ± 0.04	0.10 ± 0.05	0.11 ± 0.12
77	145 ± 8	1.81 ± 0.10	1.02 ± 0.04	0.86 ± 0.03	0.16 ± 0.03	84 ± 3	6 ± 3	0.06 ± 0.04	0.03 ± 0.02	0.13 ± 0.04	0.16 ± 0.09
85	155 ± 5	1.78 ± 0.12	1.03 ± 0.04	0.89 ± 0.06	0.14 ± 0.04	86 ± 4	5 ± 2	0.05 ± 0.02	0.04 ± 0.02	0.13 ± 0.04	0.15 ± 0.06
94	153 ± 12	1.85 ± 0.09	1.08 ± 0.06	0.91 ± 0.05	0.17 ± 0.05	84 ± 4	5 ± 3	0.05 ± 0.03	0.05 ± 0.03	0.11 ± 0.05	0.11 ± 0.10
105	163 ± 11	1.84 ± 0.15	1.12 ± 0.06	0.97 ± 0.08	0.15 ± 0.03	86 ± 3	4 ± 4	0.04 ± 0.04	0.07 ± 0.02	0.09 ± 0.05	0.06 ± 0.10
+105	165 ± 11	1.96 ± 0.12	1.20 ± 0.07	1.05 ± 0.06	0.15 ± 0.05	87 ± 4	5 ± 3	0.06 ± 0.03	0.08 ± 0.02	0.11 ± 0.04	0.09 ± 0.05
	136 ± 25	1.83 ± 0.13	1.03 ± 0.09	0.88 ± 0.09	0.15 ± 0.04	85 ± 4	6 ± 3	0.06 ± 0.03	0.05 ± 0.03	0.11 ± 0.05	0.12 ± 0.10

Values are mean ± SD.
