# Supplementary material for: Survey of Barbell Trajectory and Kinematics of the Snatch Lift from the 2015 World and 2017 Pan-American Weightlifting Championships
Source: Sports (Basel). 2020 Aug 25;8(9):118. doi: 10.3390/sports8090118 (PMC7552656; doi:10.3390/sports8090118)
Supplement: Supplementary file 1 [file sports-08-00118-s001.zip › Supplementary Table S5.pdf]

Table S5. Load and kinematic variables for heaviest successful snatch attempt for top-three women in each weight category at 2015 World Weightlifting Championship.

**Place**	**Load**	V_{max}	Y_{max}	Y_{catch}	Y_{drop}	$Catch_{rel}$	θ_1	X_1	X_2	X_{loop}	X_{net}
	**(kg)**	**(m·s^{-1})**	**(m)**	**(m)**	**(m)**	**(%)**	**(°)**	**(m)**	**(m)**	**(m)**	**(m)**
						48					
1	88	1.80	0.86	0.67	0.19	78	9	0.07	0.03	0.13	0.18
2	85	1.93	0.94	0.74	0.21	78	0	0.00	0.06	0.06	0.00
3	85	1.73	0.81	0.70	0.12	86	5	0.04	0.03	0.12	0.12
						53					
1	101	1.61	0.82	0.66	0.16	80	2	0.01	0.04	0.18	0.15
2	96	1.78	0.88	0.74	0.15	83	2	0.02	0.07	0.07	0.02
3	96	1.68	0.89	0.70	0.20	78	5	0.04	0.04	0.08	0.08
						58					
1	112	1.94	0.94	0.78	0.16	83	8	0.07	0.04	0.09	0.12
2	108	1.71	0.88	0.71	0.17	80	11	0.11	0.00	0.18	0.29
3	106	2.10	1.02	0.88	0.15	86	7	0.06	0.06	0.13	0.13
						63					
1	113	1.79	0.92	0.75	0.17	82	7	0.07	0.03	0.15	0.18
2	112	1.74	0.99	0.82	0.17	83	7	0.07	0.05	0.11	0.12
3	112	1.86	0.94	0.81	0.14	86	9	0.08	0.06	0.11	0.12
						69					
1	120	1.79	0.95	0.76	0.19	80	6	0.06	0.03	0.20	0.22
2	116	1.69	0.97	0.77	0.20	80	8	0.08	0.04	0.09	0.14
3	116	1.94	1.11	0.89	0.22	80	5	0.05	0.07	0.07	0.05
						75					
1	127	1.94	0.99	0.86	0.13	87	5	0.05	0.08	0.09	0.07
2	125	1.88	0.98	0.82	0.16	84	7	0.07	0.02	0.13	0.18
3*	-	-	-	-	-	-	-	-	-	-	-
						+75					
1	148	1.95	1.07	0.96	0.10	90	7	0.08	0.04	0.11	0.15
2	145	1.92	1.06	0.94	0.12	88	5	0.06	0.04	0.13	0.15
3	136	2.18	1.14	1.02	0.12	90	8	0.08	0.05	0.14	0.17

* Lift not recorded due to hardware/software error.
