# Supplementary material for: Survey of Barbell Trajectory and Kinematics of the Snatch Lift from the 2015 World and 2017 Pan-American Weightlifting Championships
Source: Sports (Basel). 2020 Aug 25;8(9):118. doi: 10.3390/sports8090118 (PMC7552656; doi:10.3390/sports8090118)
Supplement: Supplementary file 1 [file sports-08-00118-s001.zip › Supplementary Table S6.pdf]

Table S6. Load and kinematic variables for heaviest successful snatch attempt for top-three men in each weight category at 2015 World Weightlifting Championship.

Place	Load	V_{max}	Y_{max}	Y_{catch}	Y_{drop}	$Catch_{rel}$	θ_1	X_1	X_2	X_{loop}	X_{net}
	(kg)	($m \cdot s^{-1}$)	(m)	(m)	(m)	(%)	(°)	(m)	(m)	(m)	(m)
					56						
1	139	1.67	0.93	0.73	0.20	78	11	0.07	0.07	0.06	0.06
2	132	1.87	0.85	0.76	0.09	89	2	0.01	0.03	0.05	0.03
3	131	1.62	0.82	0.67	0.15	82	8	0.05	0.05	0.07	0.06
					62						
1	151	1.68	0.91	0.72	0.19	79	5	0.04	0.07	0.01	-0.02
2	150	1.87	0.93	0.76	0.17	82	12	0.11	0.01	0.22	0.32
3	141	1.72	0.92	0.81	0.11	88	6	0.05	0.07	0.05	0.03
					69						
1	160	1.80	0.98	0.84	0.14	86	5	0.04	0.06	0.08	0.07
2	160	1.78	0.91	0.81	0.10	89	6	0.05	0.02	0.11	0.13
3	158	1.84	0.99	0.82	0.17	83	1	0.01	0.06	0.07	0.02
					77						
1	175	1.87	1.03	0.89	0.15	86	8	0.07	0.05	0.09	0.11
2	171	1.85	1.00	0.86	0.14	86	6	0.05	0.05	0.08	0.09
3	167	1.69	0.94	0.83	0.11	88	3	0.04	0.03	0.12	0.13
					85						
1	178	1.94	1.05	0.92	0.13	87	7	0.07	0.04	0.17	0.20
2	176	1.70	0.95	0.88	0.07	93	6	0.05	0.06	0.07	0.06
3	173	1.79	0.96	0.85	0.11	88	6	0.02	0.08	0.08	0.03
					94						
1	181	2.02	1.05	0.96	0.09	91	4	0.04	0.05	0.08	0.07
2	180	1.70	1.01	0.80	0.21	79	5	0.05	0.05	0.09	0.08
3	178	1.95	1.09	0.89	0.20	82	11	0.12	0.05	0.12	0.19
					105						
1	192	1.87	1.15	0.92	0.23	80	4	0.04	0.08	0.15	0.11
2	191	1.79	1.07	0.93	0.14	87	8	0.07	0.10	0.08	0.05
3	186	1.85	1.07	0.92	0.16	85	7	0.08	0.07	0.06	0.07
					+105						
1	211	1.79	1.14	0.99	0.15	87	8	0.10	0.08	0.12	0.14
2	207	1.95	1.30	1.17	0.13	90	4	0.06	0.09	0.09	0.05
3	203	1.89	1.09	1.01	0.08	93	1	0.01	0.10	0.03	-0.05
