# Supplementary material for: Survey of Barbell Trajectory and Kinematics of the Snatch Lift from the 2015 World and 2017 Pan-American Weightlifting Championships
Source: Sports (Basel). 2020 Aug 25;8(9):118. doi: 10.3390/sports8090118 (PMC7552656; doi:10.3390/sports8090118)
Supplement: Supplementary file 1 [file sports-08-00118-s001.zip › Supplementary Table S7.pdf]

Table S7. Load and kinematic variables for heaviest successful snatch attempt for top-three women in each weight category at 2017 Pan-American Weightlifting Championship.

Place	Load	V_{max}	Y_{max}	Y_{catch}	Y_{drop}	$Catch_{rel}$	θ_1	X_1	X_2	X_{loop}	X_{net}
	(kg)	(m·s^{-1})	(m)	(m)	(m)	(%)	(°)	(m)	(m)	(m)	(m)
						48					
1	77	1.69	0.92	0.77	0.15	84	6	0.08	0.00	0.10	0.17
2	77	1.63	0.94	0.79	0.15	84	5	0.05	0.06	0.04	0.02
3*	-	-	-	-	-	-	-	-	-	-	-
						53					
1	88	1.85	1.03	0.84	0.19	82	9	0.09	0.04	0.08	0.13
2	86	1.70	0.88	0.71	0.18	80	8	0.06	0.04	0.11	0.13
3	86	1.78	0.95	0.76	0.18	81	9	0.09	0.04	0.12	0.16
						58					
1	94	1.84	0.97	0.80	0.17	83	9	0.07	0.06	0.09	0.10
2	94	1.85	1.00	0.82	0.18	82	2	0.02	0.05	0.12	0.09
3	93	1.85	0.98	0.82	0.15	85	7	0.06	0.05	0.10	0.11
						63					
1	97	2.01	1.03	0.90	0.14	87	7	0.06	0.10	0.05	0.02
2	93	1.86	0.98	0.81	0.17	82	6	0.06	0.03	0.19	0.22
3	93	2.01	1.02	0.93	0.09	91	7	0.06	0.07	0.09	0.08
						69					
1	107	1.85	0.99	0.78	0.20	80	8	0.08	0.02	0.18	0.24
2	100	1.94	1.04	0.87	0.17	84	3	0.03	0.08	0.08	0.03
3	99	2.00	1.05	0.88	0.17	84	4	0.03	0.07	0.08	0.04
						75					
1	110	1.85	1.00	0.84	0.16	84	-39	0.00	0.07	0.08	0.01
2	103	1.78	1.07	0.88	0.19	82	7	0.08	0.04	0.16	0.20
3	101	1.78	1.01	0.82	0.19	81	7	0.07	0.03	0.13	0.17
						+90					
1	120	2.16	1.24	1.14	0.10	92	7	0.07	0.10	0.11	0.09
2	119	1.86	1.17	1.02	0.15	87	11	0.15	0.05	0.24	0.34
3	118	1.92	1.11	0.99	0.11	90	6	0.07	0.08	0.12	0.11

* Lift not recorded due to hardware/software error.
