# Supplementary material for: Survey of Barbell Trajectory and Kinematics of the Snatch Lift from the 2015 World and 2017 Pan-American Weightlifting Championships
Source: Sports (Basel). 2020 Aug 25;8(9):118. doi: 10.3390/sports8090118 (PMC7552656; doi:10.3390/sports8090118)
Supplement: Supplementary file 1 [file sports-08-00118-s001.zip › Supplementary Table S8.pdf]

Table S8. Load and kinematic variables for heaviest successful snatch attempt for top-three men in each weight category at 2017 Pan-American Weightlifting Championship.

Place	Load	V_{max}	Y_{max}	Y_{catch}	Y_{drop}	$Catch_{rel}$	θ_1	X_1	X_2	X_{loop}	X_{net}
	(kg)	($m \cdot s^{-1}$)	(m)	(m)	(m)	(%)	(°)	(m)	(m)	(m)	(m)
						56					
1	119	1.61	0.86	0.75	0.11	87	6	0.05	0.03	0.12	0.14
2	118	1.70	0.87	0.80	0.07	92	6	0.05	0.00	0.14	0.19
3	108	1.85	0.95	0.79	0.16	83	6	0.06	0.01	0.17	0.22
						62					
1	125	1.71	0.92	0.81	0.11	88	8	0.07	0.02	0.13	0.18
2	124	1.71	0.91	0.75	0.15	83	11	0.10	0.03	0.06	0.13
3	123	1.85	0.95	0.78	0.18	82	4	0.03	0.05	0.04	0.03
						69					
1	145	1.93	0.99	0.90	0.09	91	10	0.11	0.01	0.16	0.25
2	143	1.65	0.96	0.79	0.17	82	5	0.05	0.04	0.13	0.14
3	137	1.71	0.98	0.82	0.16	84	5	0.06	0.03	0.12	0.15
						77					
1	162	2.00	1.02	0.81	0.20	80	8	0.08	0.01	0.15	0.22
2	152	1.69	0.99	0.84	0.15	85	10	0.11	0.02	0.14	0.23
3	151	1.77	0.95	0.82	0.13	86	8	0.09	0.01	0.23	0.31
						85					
1	160	1.78	1.05	0.88	0.17	84	7	0.08	0.01	0.19	0.25
2	159	1.77	1.07	0.90	0.17	84	3	0.03	0.03	0.17	0.17
3	158	2.00	1.09	0.99	0.10	91	4	0.04	0.06	0.08	0.06
						94					
1	169	1.93	1.05	0.89	0.16	85	8	0.08	0.03	0.11	0.16
2	166	1.86	1.07	0.87	0.20	82	4	0.04	0.02	0.18	0.20
3	166	2.01	1.24	0.99	0.25	79	6	0.06	0.07	0.05	0.03
						105					
1	182	1.78	1.13	0.97	0.16	86	2	0.02	0.05	0.10	0.07
2	175	1.93	1.12	0.98	0.13	88	4	0.05	0.09	0.06	0.02
3	167	1.63	1.15	0.94	0.21	82	2	0.02	0.04	0.08	0.07
						+105					
1	178	2.08	1.21	1.15	0.06	95	2	0.02	0.07	0.14	0.09
2	176	1.78	1.16	0.99	0.16	86	8	0.09	0.06	0.07	0.10
3	175	1.86	1.13	0.98	0.15	87	5	0.05	0.10	0.09	0.04
