# Supplementary material for: Survey of Barbell Trajectory and Kinematics of the Snatch Lift from the 2015 World and 2017 Pan-American Weightlifting Championships
Source: Sports (Basel). 2020 Aug 25;8(9):118. doi: 10.3390/sports8090118 (PMC7552656; doi:10.3390/sports8090118)
Supplement: Supplementary file 1 [file sports-08-00118-s001.zip › Supplementary Table S9.pdf]

Table S9. Results of omnibus 2x3 between-subjects ANOVAs for women.

	Competition			Placement			Competition by placement		
	**η^2**	**F**	**p**	**η^2**	**F**	**p**	**η^2**	**F**	**p**
V_{max}	0.0017	0.06	0.8	0.092	1.8	0.2	0.020	0.38	0.7
Y_{max}	0.11	4.2	0.049	0.011	0.21	0.8	0.019	0.37	0.7
Y_{catch}‡	-	-	0.3	-	-	0.4	-	-	> 0.9
Y_{drop}	0.0011	0.041	0.8	0.050	0.90	0.4	0.0036	0.066	> 0.9
$Catch_{rel}$	0.024	0.89	0.4	0.048	0.88	0.4	0.0010	0.018	> 0.9
θ_1 ‡	-	-	> 0.9	-	-	0.8	-	-	0.9
X_1 ‡	-	-	> 0.9	-	-	0.8	-	-	0.9
X_2	0.043	1.6	0.2	0.026	0.48	> 0.9	0.006	0.11	0.9
X_{loop}	0.0059	0.23	0.6	0.025	0.48	0.7	0.090	1.7	0.2
X_{net}	0.0045	0.16	0.7	0.018	0.33	0.8	0.032	0.58	0.6

‡, η^2 and F statistic were not included in robust ANOVA procedure output.
